# Mapping past land cover on Poitiers in 1993 at very high resolution using GEOBIA approach and open data

**DOI:** 10.1016/j.dib.2023.109829

**Published:** 2023-11-26

**Authors:** Elie Morin, Ny Tolotra Razafimbelo, Jean-Louis Yengué, Yvonnick Guinard, Frédéric Grandjean, Nicolas Bech

**Affiliations:** a Université de Poitiers, Laboratoire Ecologie et Biologie des Interactions (UMR CNRS 7267), 3 rue Jacques Fort, 86000 Poitiers, France; b Université de Laval, Faculté de Foresterie et Géomatique Département des sciences géomatiques, 1055 avenue du Séminaire, Québec (Québec) Canada, G1V 0A6; c Université de Poitiers, Laboratoire RURALITES, UR13823, MSHS, Bâtiment A5, 5 rue Théodore Lefèbvre, TSA 21103, 86073 Poitiers Cedex 9, France; d Grand Poitiers Communauté Urbaine, Hôtel communautaire, 84 rue des Carmélites, 86000 Poitiers, France

**Keywords:** Remote sensing, Land cover map, Landsat-5, Aerial images, GEOBIA, Poitiers

## Abstract

The land cover data presented here is a reconstruction of the past landscape (1993) at Very High Resolution (VHR) for the city of Poitiers, France. This reconstruction is based on multiple sources of images and data. We combined the strengths of both mono-temporal and multi-temporal classifications. Orthophotos were created at a spatial resolution of 0.5 m using aerial raw images from the French National Geographic Institute (IGN), taken during two aerial missions in July and August 1993. These orthophotos were merged at a spatial resolution of 5 m to conduct a first object-based classification using Landsat-5 TM images. The goal was to identify croplands, grasslands, coniferous and deciduous forests, urban areas, water bodies, and shadows. This learning-based classification employed a dataset consisting of 1371 polygons and demonstrated strong classification performances, achieving an overall accuracy of 86.31% and a kappa index of 0.832. On the other hand, mono-temporal classifications at a 0.5 m spatial resolution were carried out on each orthophoto to extract trees and herbaceous vegetation, especially in urban contexts. As mono-temporal classifications contained less information, we used a larger number of polygons for the learning step: 3849 and 5173 polygons for the northern and southern classifications, respectively. The segmentation step performed better in urban areas compared to rural areas. Consequently, the performance of classifications was evaluated separately for both contexts. Urban areas exhibited excellent performances, achieving kappa indices of 0.897 and 0.881 for the northern and southern classifications, respectively, whereas only tree vegetation was accurately detected in rural areas. To compensate for the lack of information such as buildings, railways, or roads, we modified the BD Topo^Ⓡ^ dataset from IGN. This land cover map provides highly detailed information, facilitating the understanding of urban sprawl and changes in urban and rural vegetation surrounding the city of Poitiers. Due to these reasons, this freely accessible map can be utilized by researchers, land managers, and private companies for addressing urban and ecological challenges.

Specifications Table

**Table:** 

Subject	Computer Science, Earth Sciences
Specific subject area	Remote sensing, GIS, Land Cover Map
Data format	Raw and analysed data (vector in Shapefile & Raster in format TIFF) that can be used on GIS or remote sensing software. Raster can also be opened with image readers.
Type of data	Vector and Raster
Data collection	Orthophotos were acquired from raw images of French National Geographic Institute (IGN) using the ERDAS Imagine software (https://hexagon.com/products/erdas-imagine). Landsat images were downloaded on United States Geological Survey (USGS) website (https://earthexplorer.usgs.gov/). The reference data and manual corrections were made with the QGIS software (www.qgis.org). Object-based classifications were performed using Orfeo Toolbox (www.orfeo-toolbox.org) using Random Forest classifications.
Data source location	Poitiers is an important historical metropolis of the west of France (46.34°N, 0.20°E) with 131,499 inhabitants (census in 2017). The area of the land cover map is about 225km².
Data accessibility	All data are freely available in Zenodo: https://zenodo.org/record/8220468 doi:10.5281/zenodo.8220468
Related research article	Morin, E., Herrault, P. A., Guinard, Y., Grandjean, F., & Bech, N. (2022). The promising combination of a remote sensing approach and landscape connectivity modelling at a fine scale in urban planning. Ecological indicators, 139, 108930. https://www.sciencedirect.com/science/article/pii/S1470160x22004010

## 1 Value of the Data

• The Geographic Object-Based Image Analysis (GEOBIA) method used here demonstrates the powerful combination of i) multi-temporal classification using satellite images to differentiate croplands from grasslands for example and ii) mono-temporal classification to recover small objects like hedgerows or isolated tree in urban and rural areas.
• This land cover map is valuable for Poitiers that have been experienced a strong expansion of its urban areas since 1993 showing now a High-Speed-Railway, more housing areas, larger industrial areas, and larger surrounding towns. Such a map allows to monitor the landscape through time, representing thus a useful data in land management.
• As land cover changes is crucial to land management, this map will help to understand changes from 1993 to now for urban, agricultural issues but also their impact on ecological processes. Data will be easily used in GIS applications for any users.

## 2 Data Description

**Fig. 1:**
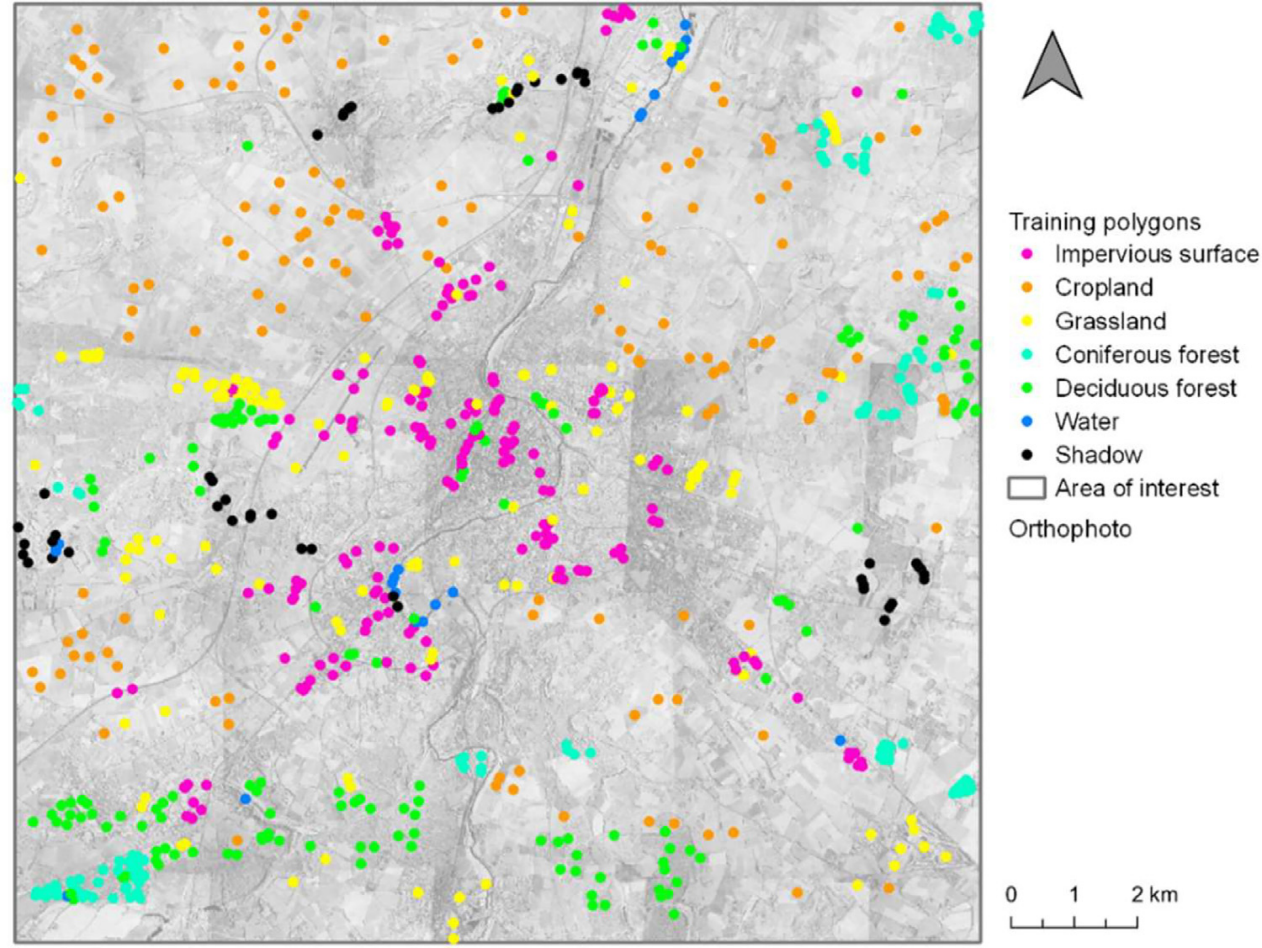
Distribution of the training polygons for the multi-temporal classification.

**Fig. 2:**
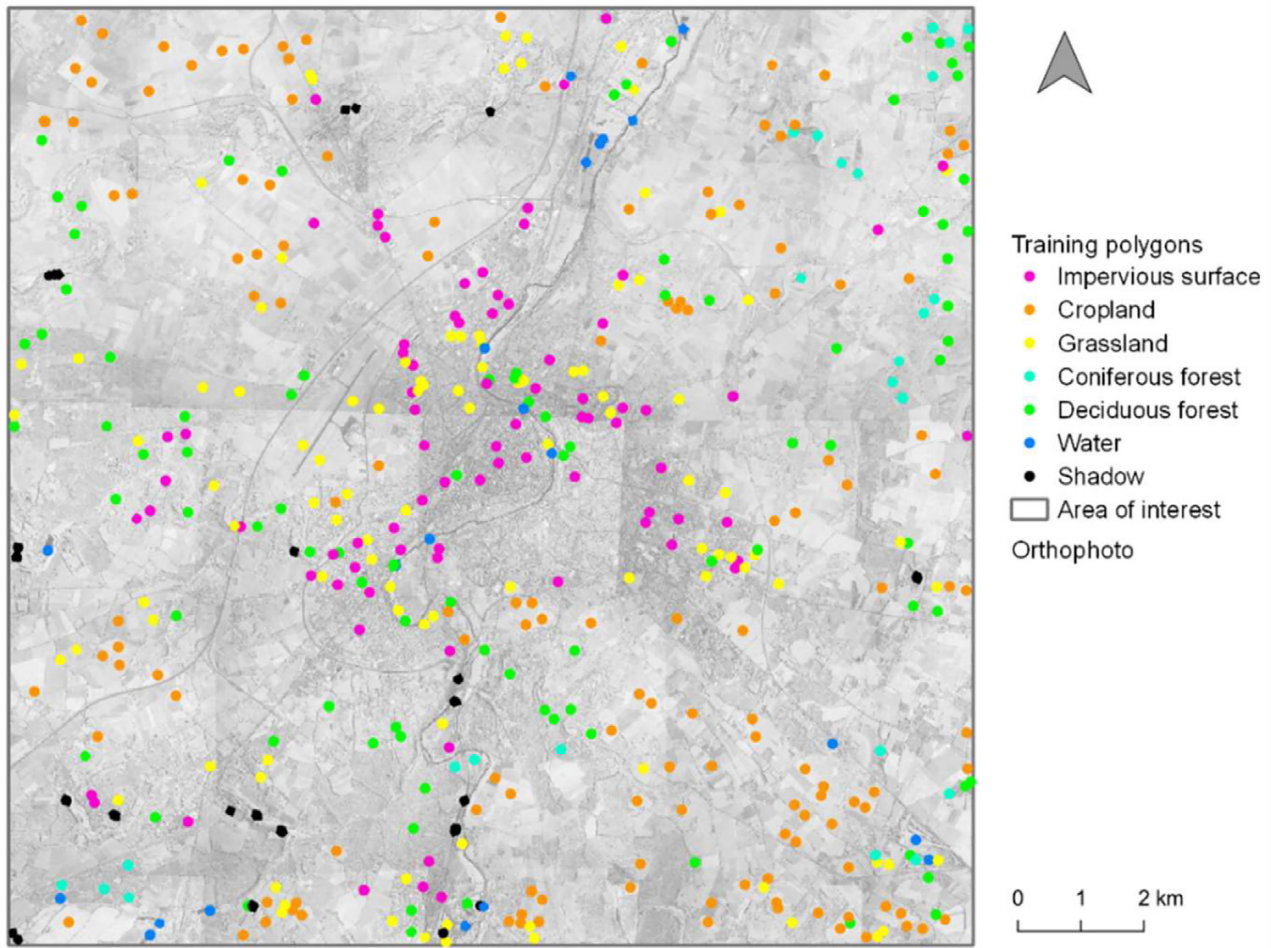
Distribution of the validating polygons for the multi-temporal classification.

**Fig. 3:**
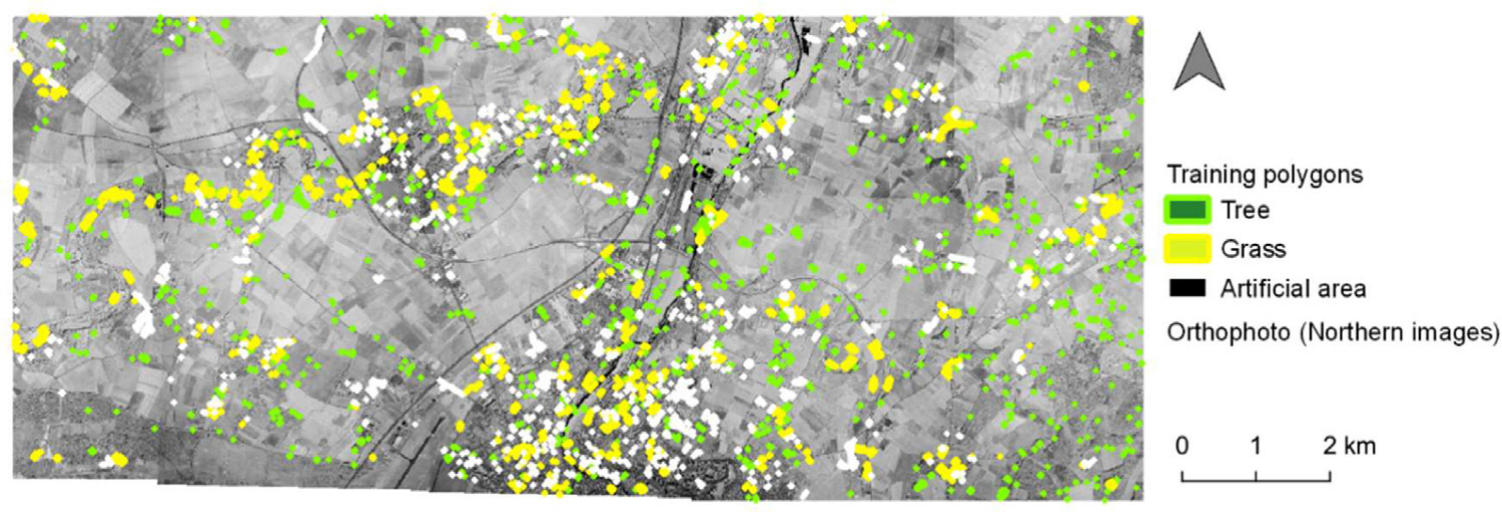
Distribution of the training polygons for the mono-temporal classification on northern images.

**Fig. 4:**
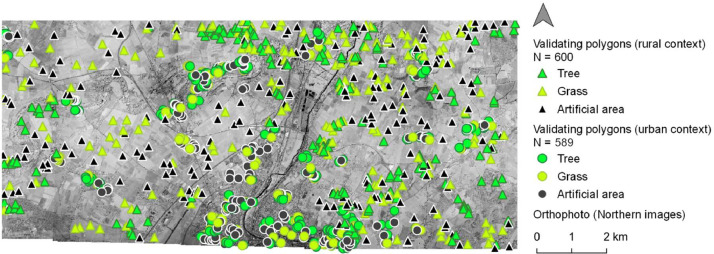
Distribution of the validating polygons for the mono-temporal classification on northern images.

**Fig. 5:**
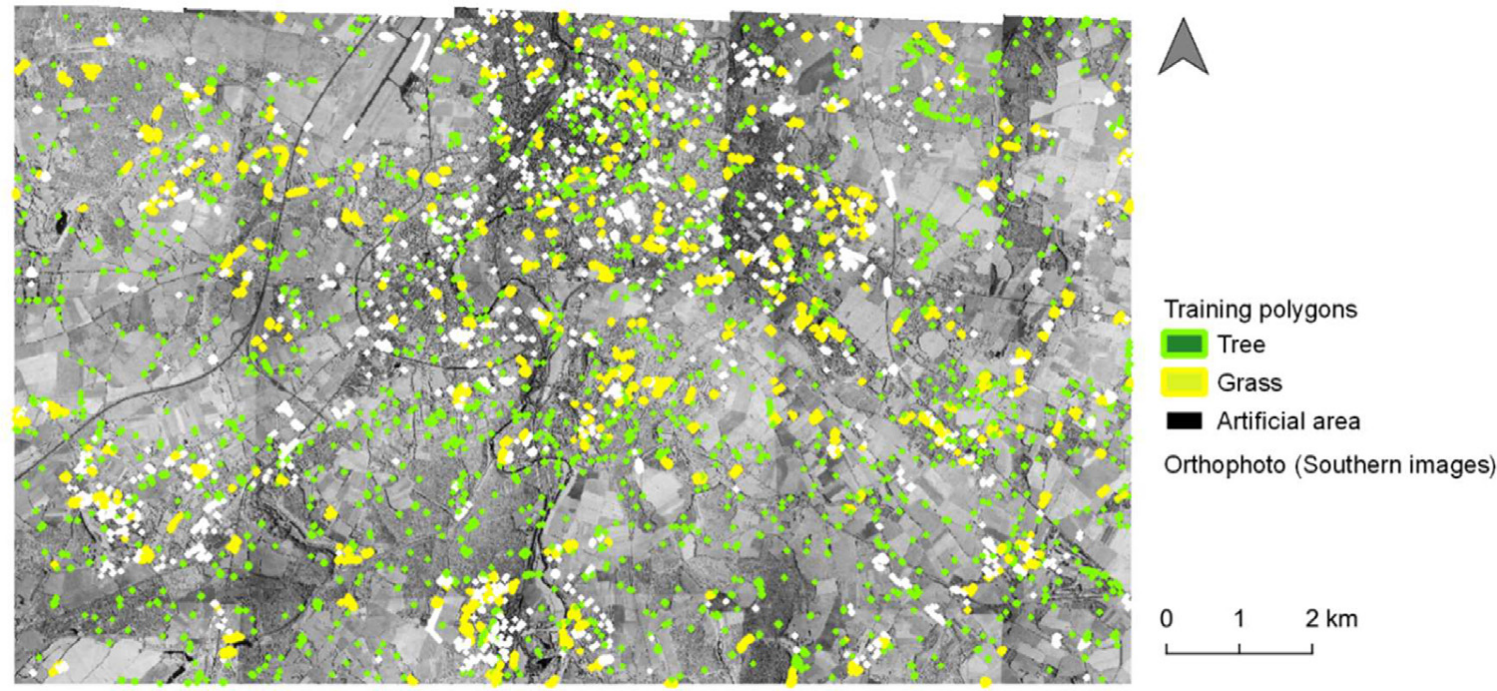
Distribution of the training polygons for the mono-temporal classification on southern images.

**Fig. 6:**
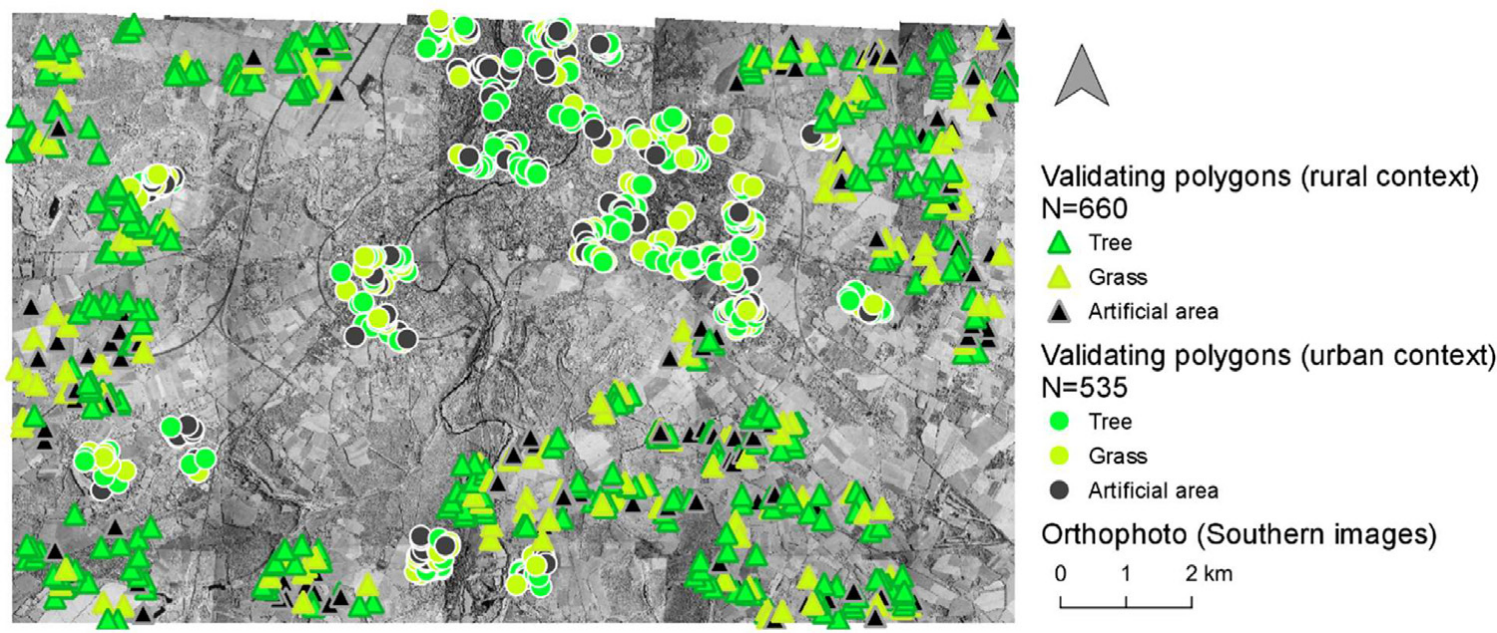
Distribution of the validating polygons for the mono-temporal classification on southern images.

The land cover analysis of Poitiers in 1993 used aerial images at a Very High Resolution (VHR) and Landsat-5 TM images at High Resolution (HR). In addition, training and validating polygons were created to conduct learning-based classifications (i.e., Random Forest classification) et evaluate their performance (Fig. 1, Fig. 2, Fig. 3, Fig. 4, Fig. 5, Fig. 6).

Digital aerial images were provided by the IGN, contained a NIR, red and green channel and were acquired in summer 1993 over the area of interest. They were acquired during two different missions: 20 tiles for the north of Poitiers were taken on 16 August 1993 and 30 tiles for the south of Poitiers were taken on 28 July 1993 (Table 1). It is important to note that different date acquisition can influence spectral information due to phenological variation of the vegetation. Orthorectification was conducted on each tile using ERDAS IMAGINE version 2015 (Appendix A). The images were projected into the Lambert-93 (EPSG: 2154) projection based on the RGF93 geoid (IAG GRS 1980 ellipsoid) and were resampled to a spatial resolution of 0.5 m. Then, mosaics were performed from these orthorectified tiles for each mission (Northern and Southern images). The topographic correction, radiometric calibration, atmospheric and anisotropy correction were not performed because the needed parameters (e.g., atmospheric composition, aerosol types) were not available. Even though the pre-processing step is not optimal as in many other conditions or studies, the main objective was to get reliable information to conduct a classification on these images.

**Table 1:** Characteristics of used images.

Type	Date	Path	Row	Spatial resolution	Spectral resolution
Orthophoto (Northern images)	08/16/1993	/	/	0.5 m	G/R/NIR
Orthophoto (Southern images)	07/28/1993				
Landsat 5 TM	01/22/1992	200	28	30 m	B/G/R/NIR/MIR
Landsat 5 TM	02/23/1992				
Landsat 5 TM	03/10/1992				
Landsat 5 TM	04/11/1992				
Landsat 5 TM	05/13/1992				
Landsat 5 TM	07/16/1992				
Landsat 5 TM	08/20/1993				

We used multi-temporal Landsat scenes to catch variations of vegetation. Landsat images have been widely used to consider the multi-seasonal variation of spectral information of vegetation due to the different phenology of plant species [18,23]. We used seven Landsat Thematic Mapper 5 scenes acquired between January 1992 and August 1993 (Table 1) to differentiate, for example, cropland from grassland or deciduous from coniferous. The satellite images were cloud-free and were converted to reflectance values and rescaled from 0 to 255 (8 bits) for each spectral band.

The OTB open-source project [9] was used to conduct the remote sensing analyses and QGIS project [17] was used for the remote sensing and GIS analyses.

## 3 Experimental Design, Material and Methods

### 3.1 Steps of multi-temporal classification

Two classifications were conducted successively using multi-temporal data to get the main land cover categories and using mono-temporal data to extract small vegetation elements such as isolated trees or hedgerows. To identify the main objects such as impervious areas, forests, grasslands, or croplands, the two orthophotos were merged and resampled to a spatial resolution of 5 m using bilinear method to facilitate the segmentation. The following algorithms were used from the OTB project [9].

#### 3.1.1 Segmentation

The segmentation is an important step of the object-based classification as its results influence the classification accuracy ([8]; Jian [11,21]). The objective is to create groups of spatially close pixels with similar radiometric characteristics [1,4]. We used the *MeanShiftSegmentation* algorithm, this procedure contains four steps: i) a smoothing step facilitating the segmentation, ii) the segmentation step creating segments, iii) the merging step that fusion small segments to similar neighbour objects, iv) the vectorization step to convert pixel segments into vector segments and calculate mean and standard deviation of each raster band within segments [9]. The spatial radius and the range radius manage the smoothing effect and the capacity to preserve edges between objects. These parameters should not be too high to avoid under-segmentation and not too low to avoid over-segmentation. The minimum segment size avoids very small objects. In addition, the spatial resolution of the input image impacts the segmentation step. For the *LargeScaleMeanShift* algorithm, we selected a spatial radius of 5 pixels, a range radius of 15 pixels and a minimum segment size of 12 pixels.

#### 3.1.2 Feature extraction

The feature extraction allows to distinguish categories during the classification step. Thus, segments must contain information related to spectral, texture, 3D or geometric characteristics [15,20]. For each segment, we computed the mean and standard deviation value of the NIR, Red and Green channel of the orthophoto, the mean value of raster bands of Landsat images (i.e., Blue, Green, Red, NIR, MIR channel) (Appendix B). To improve the detection of water, the modified normalized difference water index (MNDWI) [22] was calculated for the 7 Landsat scene and their mean value were computed within segments. Moreover, the number of pixels of each segment was extracted for the classification step.

#### 3.1.3 Random forest classification

Random forest has been introduced by Breiman [2] as an ensemble of classification methods. This approach uses classification and regression trees (CART; [3]) where each tree gives a classification to identify the most prevalent class. Then, an out-of-bag (OOB) error is calculated to estimate the rate of misclassified samples. This learning approach is widely used in remote sensing classification for its capacity to manage many variables, its stability and robustness. Moreover, there is few parameters to tune which are i) the number of trees within the forest and ii) the depth of each tree. The number of trees in the forest was set to 250 and the depth of tree to 7, corresponding to the square root of the number of variables (i.e., 49; Appendix B) [18].

#### 3.1.4 Training and validating polygons

The selection of training and validating polygons is crucial for the classification accuracy. The best classification accuracy is obtained when statistics of categories show low intra-class variation and high inter-class variation. Thus, they must represent statistical variation within each class. Training and validating polygons were chose across all the studied area, to avoid spatial autocorrelation, which can have an influence on the evaluation of classification accuracy (Fig. 1; [12]). From the segmentation, we selected segments by photointerpretation (based on the orthophotos and Landsat images) to construct the training dataset. Seven target classes were identified for the classification: Impervious surface contained buildings, roads, bare soils; cropland contained annual crops; grassland contained meadows and herbaceous vegetation; coniferous forest; deciduous forest; water contained the main water bodies and courses; and shadow (Table 2). The number of polygons was roughly based on the relative importance of each category considering the classification objectives.

**Table 2:** Training dataset used for the multitemporal classification.

Dataset	Category	Number of polygons
Training dataset		
	Impervious surface	195
	Cropland	156
	Grassland	160
	Coniferous	152
	Deciduous	157
	Water	23
	Shadow	49
	Total	892
Validating dataset		
	Impervious surface	80
	Cropland	137
	Grassland	101
	Coniferous	25
	Deciduous	91
	Water	21
	Shadow	24
	Total	479

The evaluation of classification accuracy is based on the number of pixels well assigned. Thus, we designed validating polygons with similar area corresponding to approximately 12 pixels (Fig. 2; [18]). To evaluate the global performance of the classification, the overall accuracy and the kappa index were computed. Three metrics were also calculated at the class-level: the precision (also called Producer's accuracy), the recall (also called User's accuracy) and the F-Score (which is the harmonic mean of the precision and the recall).

### 3.2 Steps of mono-temporal classifications

We conducted classifications at VHR from orthophotos to a 0.5 m of spatial resolution using a similar workflow as previously. However, each of the original orthophoto were treated separately because of their spectral differences induced by their acquisition condition, in order to increase the classification performances. Conversely, the main information for the multi-temporal classification was contained in the Landsat images, so the differences between the two orthophotos were negligible. Consequently, two classifications were realized (i.e., Northern orthophoto & Southern orthophoto). Due to the nature of the input data, this procedure was highly similar to the method proposed by Morin et al. [14]. This approach was mainly developed for highly heterogeneous and complex landscapes such as the urban environment.

#### 3.2.1 Segmentation

This segmentation step aims to create appropriate segments for small objects such as the canopy of a tree. For the *LargeScaleMeanShift* algorithm, we selected a spatial radius of 5 pixels, a range radius of 28 pixels and a minimum segment size of 60 pixels. A large value of the range radius induces a strong smooth effect facilitating the segmentation that is particularly interesting for very high resolution images. Moreover, the minimum size of 60 pixels is a good compromise for detecting isolated trees and avoiding oversegmentation.

#### 3.2.2 Feature extraction

There are some limits to conduct a classification only from the spectral information of a mono-temporal image. For example, the absence of the height of each object can impedes the identification of vegetation strata. However, textures have been used with success to distinguish vegetation strata or urban objects [5,^7^,13]. Textures have been described as the smoothness, the regularity, or the coarseness of an image (Gonzalez & Woods 2002). This aspect of an image can be statistically captured from a grey level co-occurrence matrix [10,16] that represent the spatial homogeneity around the pixels using a specific size of moving window. The matrix quantifying the variation in grey levels is then used to compute the textures. Thus, spectral and texture information were calculated to improve the classification accuracy. The Normalized Difference Vegetation Index (NDVI) [19] was computed, a widely used index to detect vegetation in remote sensing applications. The brightness was calculated as the mean of NIR, Red and Green band. In addition, four Haralick's textures were computed from the brightness using the *HaralickTextureExtraction* algorithm with a window size of 7 × 7 pixels: *Energy, Entropy, Correlation, Contrast*. The average of the NIR, Red, Green, the NDVI and the four texture was included within segments as well as the standard deviation of the Green and the four textures (Appendix B). Segments were filtered before the classification to remove the shadow segments which showed a brightness value lower than 95 for the Northern orthophoto and 80 for the Southern orthophoto.

#### 3.2.3 Random forest classification

We used the Random Forest algorithm for the two classifications. For the parameter values of the *TrainVectorClassifier* algorithm, we chose a value of 250 for the number of trees and a value of 4 for the depth of tree (i.e., the square root of the number of the input variables) [18]. A stratified subsampling followed by a cross-validation process was also used by dividing the training dataset into five roughly equal subsets to conduct 5 different classifications using the *VectorClassifier* algorithm [6,14]. Then, these classifications were merged with a majority voting (i.e., *FusionOfClassifications* algorithm). The overall accuracy and the Kappa index were computed to estimate the global performance of the two classifications. The precision, recall and F-Score were calculated for each land cover category.

#### 3.2.4 Training and validating polygons

The herbaceous vegetation, the wooded vegetation and the artificial area were the three target categories. Because less information was available compared to the first classification that used a multi-temporal approach, a cross validation process with a larger number of training segments were chose (Table 3; Figs. 3, 5). To estimate the accuracy of each classification, we randomly designed validating polygons with similar area corresponding to approximately 60 pixels (Figs. 4, 6; [18]). As this method was optimized for urban context but was conducted on the whole area of interest in this paper, we evaluated the accuracy of the two classifications in urban context and rural context separately and together.

**Table 3:** Training and validating polygons used for each mono-temporal classification.

Training polygons		
Orthophoto	Category	Number of polygons
Northern images	Artificial area	882
	Herbaceous vegetation	875
	Wooded vegetation	903
	Total	2660
Southern images	Artificial area	1252
	Herbaceous vegetation	1081
	Wooded vegetation	1645
	Total	3978

Validating polygons		
Orthophoto	Category	Number of polygons
Northern images		
Rural context	Artificial area	200
	Herbaceous vegetation	200
	Wooded vegetation	200
	Total	600
Urban context	Artificial area	216
	Herbaceous vegetation	148
	Wooded vegetation	225
	Total	589
		
Urban & rural context	Artificial area	416
	Herbaceous vegetation	348
	Wooded vegetation	425
	Total	1189
Southern images		
Rural context	Artificial area	125
	Herbaceous vegetation	144
	Wooded vegetation	391
	Total	660
Urban context	Artificial area	130
	Herbaceous vegetation	195
	Wooded vegetation	210
	Total	535
Urban & rural context	Artificial area	255
	Herbaceous vegetation	339
	Wooded vegetation	601
	Total	1195

### 3.3 Segmentation results for the two different approaches

**Fig. 7:**
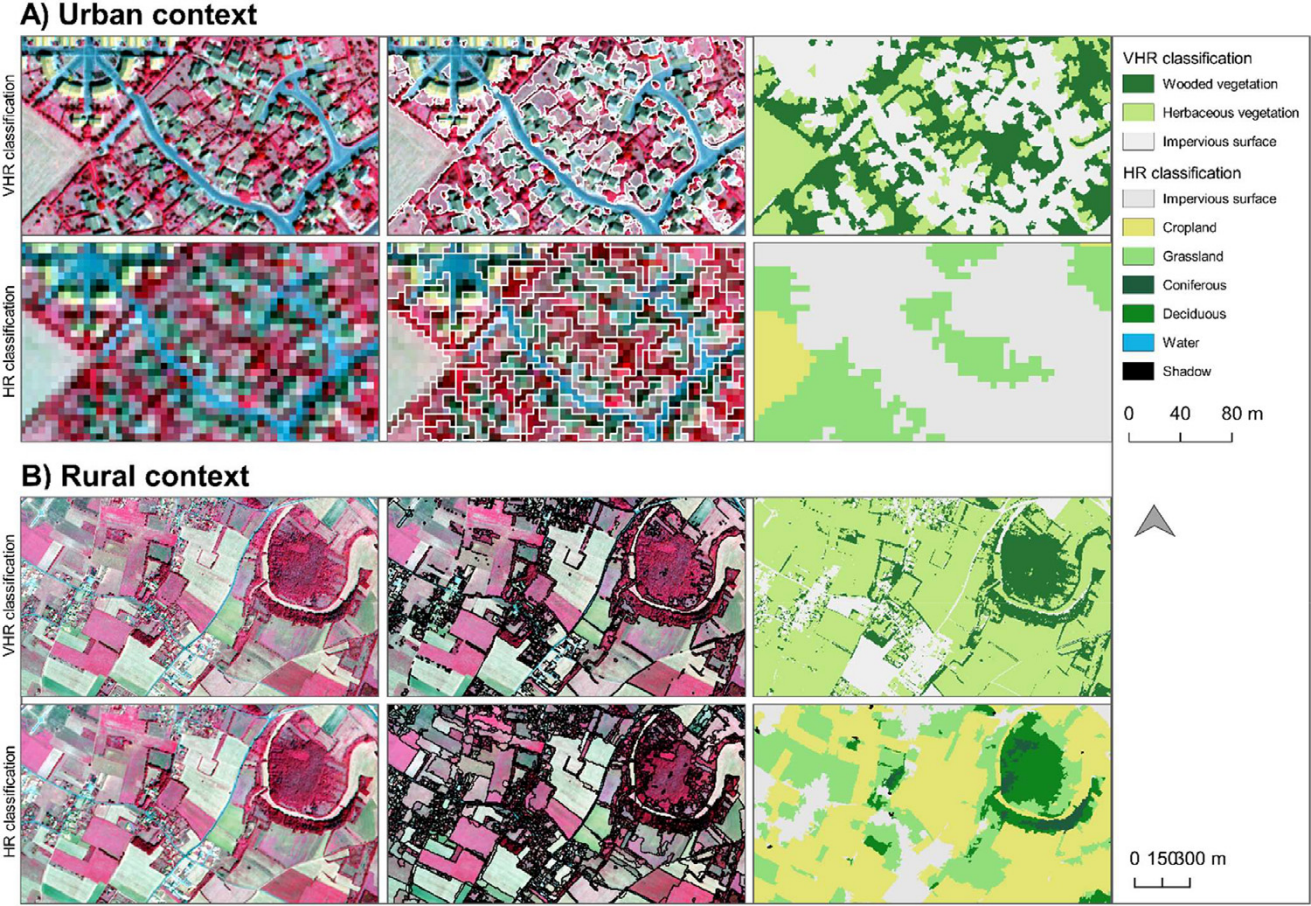
Results of segmentation and classification at both scales.

The segmentation is an important process to create semantic objects. The two segmentations were conducted from the same mono-temporal images but at two different spatial resolutions (i.e., 0.5 m and 5 m). Results showed that the resolution and the chosen parameters influenced the created segments. Differences rely on the complexity and heterogeneity of the environment. In rural areas where heterogeneity and complexity are low, segmentation at HR was more relevant to delimitate crops (Fig. 7). Conversely in urban areas which are very complex and heterogeneous landscapes, VHR segmentation well performed to identify artificial from tree and grass objects (Fig. 7). The segmentation at HR produced 359,750 segments for the whole area of interest and the segmentations at VHR produced 710,688 segments for the Northern images and 1514,964 for the Southern images.

### 3.4 Analysis of accuracy assessments

The multi-temporal classification showed good classification results with an overall accuracy and a kappa index of 86,31% and 0.832 respectively. Regarding the accuracy at the class-level, impervious surface, cropland and shadow were well identified with high F-Score (i.e., around 90%) (Table 4). Grassland showed relatively high F-Score but had confusions with impervious surface (in urban context) and deciduous forest (mainly due to meadows close to forest and for which Landsat pixel overlapped) (Table 4). Deciduous forest was well detected but had some true deciduous pixels classified in other categories (recall value of 77%) such as coniferous forest or grassland. Coniferous forest was overestimated by having a low precision value and showing a confusion with deciduous forest and water and well classified true coniferous pixels (i.e., recall value of 100%). The water category was underestimated with a good precision value but a low recall value identifying a confusion mainly with the impervious surface category (Table 4).

**Table 4:** Confusion matrix of the multitemporal classification: impervious surface (1), cropland (2), grassland (3), coniferous forest (4), deciduous forest (5), water (6), shadow (7).

	Ground truth								Recall (%)
		1	2	3	4	5	6	7	
Classified	**1**	992	0	0	0	0	22	0	97.83
	**2**	13	1665	128	0	0	0	0	92.19
	**3**	67	142	981	0	66	0	0	78.10
	**4**	0	0	0	316	0	0	0	100
	**5**	13	0	67	180	921	0	0	77.98
	**6**	115	0	0	4	0	169	11	56.52
	**7**	0	0	0	51	4	0	526	90.53
	Precision (%)	82.66	92.14	83.41	57.35	92.93	88.48	97.95	
	F-Score (%)	89.61	92.17	80.67	72.90	84.81	68.98	94.10	

It was more contrasted for the VHR classifications. The accuracy of the northern classification was quite low, considering only three land cover categories, with an overall accuracy of 80.74% and a kappa index of 0.713 for the northern classification but quite better for the southern classification with an overall of 88.36% and a kappa index of 0.814. However, the use of independent validating polygons in rural and urban areas allows to estimate the accuracy of both classifications in these different contexts.

In urban context, the classification accuracy was high with an overall accuracy of 93.20% and kappa index of 0.897 for the northern classification and an overall accuracy of 92.20% and a kappa index of 0.881 for the southern classification. Conversely, in rural context results were lower with an overall accuracy of 68.49% and a kappa index of 0.527 for the northern classification and an overall accuracy of 85.25% and a kappa index of 0.746 for the southern classification. Wooded vegetation was well detected in both context by showing high F-Score values (Table 5, Appendix C).

**Table 5:** Performances of both classifications evaluated in urban context, rural context and urban-rural context.

Northern classification				
Context	Categories	Precision (%)	Recall (%)	F-Score (%)
Urban				
	Wooded vegetation	96.29	89.23	92.63
	Herbaceous vegetation	86.05	91.33	88.61
	Impervious surface	95.34	98.62	96.95
Rural				
	Wooded vegetation	96.46	84.58	90.13
	Herbaceous vegetation	52.13	89.93	66.01
	Impervious surface	77.93	30.94	44.3
Global				
	Wooded vegetation	96.37	87.05	91.47
	Herbaceous vegetation	62.75	90.53	74.12
	Impervious surface	90.77	66.08	76.48

Southern classification				
Context	Categories	Precision (%)	Recall (%)	F-Score (%)
Urban				
	Wooded vegetation	96.83	90.2	93.4
	Herbaceous vegetation	88.22	93.78	90.91
	Impervious surface	91.58	93.07	92.32
Rural				
	Wooded vegetation	99.47	91.97	95.57
	Herbaceous vegetation	61.43	93.94	74.28
	Impervious surface	86.64	54.3	66.76
Global				
	Wooded vegetation	98.54	91.35	94.81
	Herbaceous vegetation	74.4	93.85	83.01
	Impervious surface	89.74	74.05	81.14

### 3.5 Compiling data

**Fig. 8:**
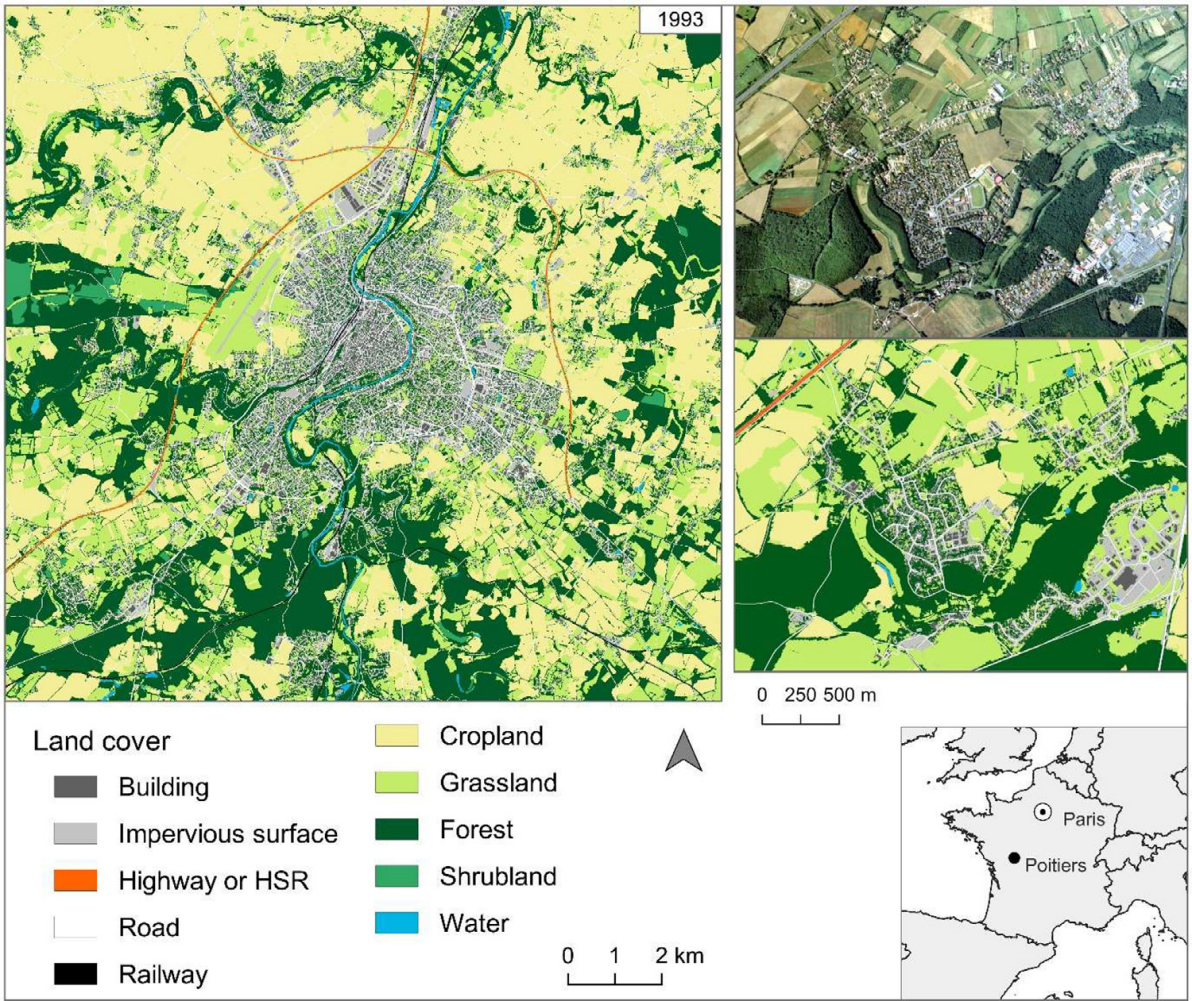
Land cover map of Poitiers in 1993.

We compiled the created data and available institutional databases to build a map at very high spatial resolution (Table 6; Fig. 8). Buildings, roads, railways and water courses and bodies were manually modified from existing data (BD Topo of IGN) by photointerpretation. Deciduous and coniferous forests, croplands, grasslands and artificial areas were extracted from the multi-temporal classification. Water from the HR classification was not used due to its coarse resolution and because this category was mainly integrated into the classification to not overestimate the impervious surfaces which could have close radiometric information. Urban vegetation strata, detected within the artificial areas of the multi-temporal classification, were extracted from the mono-temporal classifications. In addition, the wooded vegetation of mono-temporal classifications was also integrated in rural areas du to its good detection. Shrublands were added by creating polygons using photointerpretation from orthophotos.

**Table 6:** Databases used to build the land cover map of 1993.

Land cover	Database
Building	Existing database (updated BD Topo - IGN)
Impervious surface	Multi-temporal classification
Road	Existing database (updated BD Topo - IGN)
Railway	Existing database (updated BD Topo - IGN)
Cropland	Multi-temporal classification
Grassland	Multi-temporal classification
Urban herbaceous vegetation	Mono-temporal classifications (VHR)
Forest	Multi-temporal classification
Rural wooded vegetation (e.g., hedgerows)	Mono-temporal classifications (VHR)
Urban wooded vegetation	Mono-temporal classifications (VHR)
Shrubland	Mono-temporal classifications (VHR)
Water	Existing database (updated BD Topo - IGN)

## Limitations

Despite the high accuracy of the land use map, shrub areas are difficult to classify and can be classified as either tree or herbaceous vegetation.

## Ethics Statement

The authors have read and follow the ethical requirements for publication in Data in Brief and confirmed that the current work does not involve human subjects, animal experiments, or any data collected from social media platforms.

## CRediT authorship contribution statement

**Elie Morin:** Conceptualization, Methodology, Software, Formal analysis, Investigation, Data curation, Writing – original draft, Writing – review & editing, Visualization, Validation. **Ny Tolotra Razafimbelo:** Software, Formal analysis. **Jean-Louis Yengué:** Project administration, Funding acquisition. **Yvonnick Guinard:** Supervision, Project administration, Funding acquisition. **Frédéric Grandjean:** Writing – review & editing, Project administration, Supervision, Funding acquisition. **Nicolas Bech:** Conceptualization, Methodology, Writing – review & editing, Validation, Supervision, Project administration, Funding acquisition.

## Figures and Tables

**Fig. A1:**
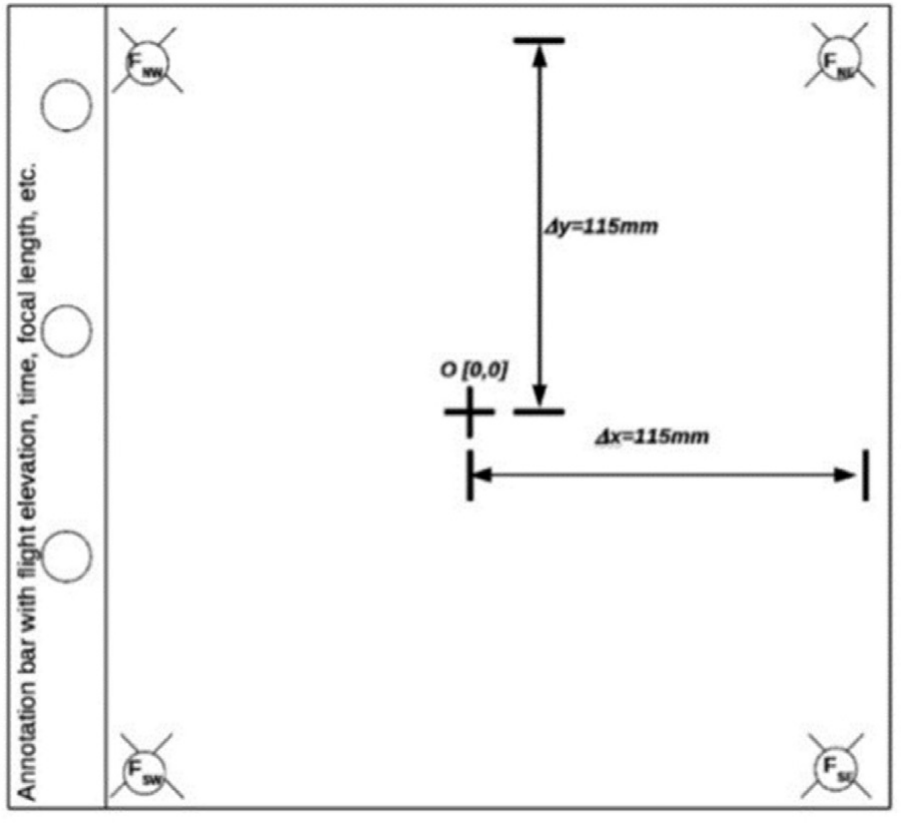
Graphic representation of diagonal fiducial marks (F) on a 230 mm* 230 mm aerial photograph, with the following coordinates in mm in the photo coordinate system: FNE[115, 115], FSE[115, 115], FSW[115, 115], and FNW[115, 115]. (Rocchini et al., 2012).

**Fig. A2:**
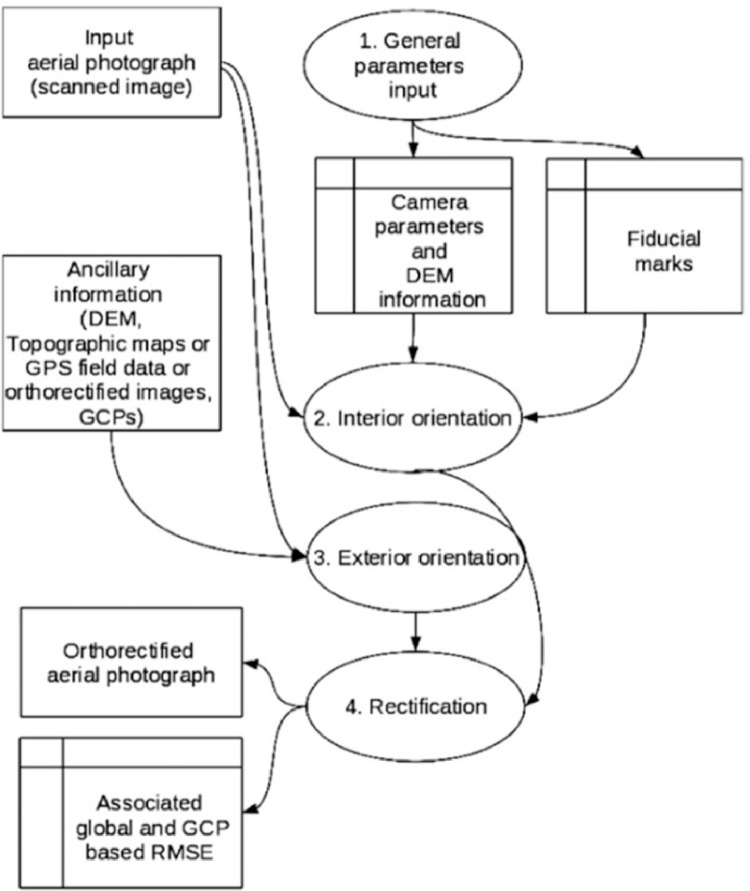
General flow of the orthorectification procedure (Rocchini et al., 2012).

**Table:** 

Raster	Source of information	Information type	Mean	SD
Nb pixels		Geometric		
Green	Orthophoto	Spectral	X	X
Red	Orthophoto	Spectral	X	X
NIR	Orthophoto	Spectral	X	X
Blue	Landsat (x 7 images)	Spectral	X	
Green	Landsat (x 7 images)	Spectral	X	
Red	Landsat (x 7 images)	Spectral	X	
NIR	Landsat (x 7 images)	Spectral	X	
MIR	Landsat (x 7 images)	Spectral	X	
MNDWI	Landsat (x 7 images)	Spectral	X	

**Table:** 

Raster	Information type	Mean	SD
NIR	Spectral	X	
Red	Spectral	X	
Green	Spectral	X	X
NDVI	Spectral	X	
Energy	Texture	X	X
Entropy	Texture	X	X
Correlation	Texture	X	X
Contrast	Texture	X	X

**Table:** 

Land cover categories are coded as following : 1 = impervious surface, 2 = cropland, 3 = grassland, 4 = coniferous forest, 5 = deciduous forest, 6 = water, 7 = shadow									
	Ground truth								Recall (%)
		1	2	3	4	5	6	7	
Classified	**1**	992	0	0	0	0	22	0	97.83
	**2**	13	1665	128	0	0	0	0	92.19
	**3**	67	142	981	0	66	0	0	78.1
	**4**	0	0	0	316	0	0	0	100
	**5**	13	0	67	180	921	0	0	77.98
	**6**	115	0	0	4	0	169	11	56.52
	**7**	0	0	0	51	4	0	526	90.53
	Precision (%)	82.66	92.14	83.41	57.35	92.93	88.48	97.95	
	F-Score (%)	89.61	92.17	80.67	72.9	84.81	68.98	94.1	
Overall accuracy = 86.31%									
Kappa index = 83.22%									

**Table:** 

Land cover categories are coded as following : 1 = Wooded vegetation, 2 = Herbaceous vegetation, 3 = impervious surfaces				
Northern images				
Urban context	Ground truth			
		1	2	3
Classified	**1**	22,698	2205	532
	**2**	809	15,272	640
	**3**	65	270	24,008
Overall accuracy = 93.20%				
Kappa index = 89.67%				

Rural context	Ground truth			
		1	2	3

Classified	**1**	19,067	3073	403
	**2**	699	20,304	1573
	**3**	0	15,570	6978
Overall accuracy = 68.49%				
Kappa index = 52.73%				

Urban & rural context	Ground truth			
		1	2	3
Classified	**1**	41,765	5278	935
	**2**	1508	35,576	2213
	**3**	65	15,840	30,986
Overall accuracy = 80.74%				
Kappa index = 71.33%				

Southern images				
Urban context	Ground truth			
		1	2	3
Classified	**1**	21,372	1861	460
	**2**	569	20,587	796
	**3**	130	887	13,665
Overall accuracy = 92.20%				
Kappa index = 88.10%				
Rural context	Ground truth			
		1	2	3

Classified	**1**	40,589	3148	395
	**2**	197	15,278	788
	**3**	18	6443	7678
Overall accuracy = 85.25%				
Kappa index = 74.60%				

Urban & rural context	Ground truth			
		**1**	**2**	**3**
Classified	**1**	61,961	5009	855
	**2**	766	35,865	1584
	**3**	148	7330	21,343
Overall accuracy = 88.36%				
Kappa index = 81.42%				

## Data Availability

Mapping past land cover on Poitiers in 1993 at Very High Resolution using GEOBIA approach and open data (Original data) (Zenodo).

## Appendix A

We used the method of geometric modelling based on the collinearity equation and implemented in the Leica Photogrammetry Suite (integrated in ERDAS Imagine). The ERDAS Imagine software is a simple and useful tool to visualize and manipulate geographic imagery data. The ERDAS Imagine software can perform a wide variety of tasks such as orthorectification (Long & Srihar, 2004) for which it is considered the most reliable. Moreover, during orthorectification, the software incorporates the following parameters:

(i) The reference system in which the image will be georeferenced,
(ii) The Digital Terrain Model (DTM) used to derive the elevation of the Ground Points Control (GCP),
(iii) The camera and raw image parameters (see Fig. A1):

- the focal length in millimeters,

- the coordinates in millimeters in the system of the photo of the main point of symmetry, which symmetry point, which are ideally [0,0] but not necessarily,

- the number of fiducial points used for the interior orientation,

-the coordinates of the fiducials in millimeters in the coordinate system of the photo.

According to Rocchini et al., 2012, The ortho-rectification steps (Fig. A2) include:

• **Interior orientation:** The interior orientation of the photograph is needed to relate the image coordinate system (in pixels) to the photo coordinate system (camera sensor). This is done by graphically marking the fiducial marks into the scanned image.
• **Exterior orientation:** Exterior orientation of the aerial photograph is based on the selection of n GCPs, taking into account their x and y (coordinates of each GCP of the incorrect input image in the image coordinate system), their x0 and y0 (corresponding GCP coordinates of the rectified image in the target coordinate system defined by projection parameters), and z coordinates.
• **Rectification and resampling:** The parameters computed during the interior and exterior orientation steps are used to rectify the image, ultimately passing from the input x, y image coordinates to the x0, y0 standard map coordinates in the target projection.

Finally, after having retrieved the parameters from the IGN and the DTM, we have orthorectified the 50 tiles taken in 1993 in the surroundings of Poitiers. We have, compiled these tiles into two mosaics (corresponding to the two periods in which the photos were taken: July in the South and August in the North). We trimmed these two mosaics in order to obtain a global study area of 15 km by 15 km.

### Bibliography

Long, W., & Srihar, S. (2004). Land cover classification of SSC image: Unsupervised and supervised classification using ERDAS imagine. *International Geoscience and Remote Sensing Symposium (IGARSS), 4*, 2707–2712. https://doi.org/10.1109/igarss.2004.1369859.
Rocchini, D., Metz, M., Frigeri, A., Delucchi, L., Marcantonio, M., & Neteler, M. (2012). Robust rectification of aerial photographs in an open source environment. *Computers and Geosciences, 39*, 145–151. https://doi.org/10.1016/j.cageo.2011.06.002.

## Appendix B

Features extracted and used for the multi-temporal classification.

Features used for the mono-temporal classifications.

## Appendix C

**Multi-temporal classification**

**Mono-temporal Classifications**
